# Effect of dietary histamine supplementation on growth, digestive enzyme activities and morphology of intestine and hepatopancreas in the Chinese mitten crab *Eriocheir sinensis*

**DOI:** 10.1186/s40064-016-2105-9

**Published:** 2016-04-30

**Authors:** Liulan Zhao, Xiaozhen Yang, Yongxu Cheng, Song Yang

**Affiliations:** Key Laboratory of Exploration and Utilization of Aquatic Resources and Aquaculture Division, Life Science and Aquaculture College, Shanghai Ocean University, No. 999 Huchenghuan Road, Lingang New District, Shanghai, 201306 People’s Republic of China; College of Animal Science and Technology, Sichuan Agricultural University, Chengdu, Sichuan Province 611130 People’s Republic of China

**Keywords:** Histamine, Growth, Digestive enzyme, Morphology, Intestine, Hepatopancreas, *Eriocheir sinensis*

## Abstract

A 28-days feeding experiment was conducted to investigate the effect of histamine on digestive physiology of the Chinese mitten crab, *Eriocheir sinensis*. Four experimental diets were supplemented with histamine at 0, 1, 2, 4 g/kg. Histamine supplementation had no effect on growth. The activities of digestive enzyme decreased significantly at first (days 7 and 14) (*p* < *0*.*05*) and then increased or finally slightly recovered in the hepatopancreas and intestinal tract on the 28th day. Tryptase and amylase activities were inhibited significantly in each histamine-treated group on day 7 as compared to the control (*p* < *0*.*05*). On day 7, 14 and 28, tryptase mRNA relative expression in the histamine treatments correlated positively with the histamine concentration (*p* < *0*.*05*). Histopathologic analyses showed serious alterations in hepatopancreas, moderate alterations in the hindgut and intestinal bulb, and no alterations in the midgut. In hepatopancreas, low levels (1 g/kg) of histamine caused an increase in the number of B-cells. High levels (4 g/kg) of histamine increased the number of R-cells, which were also highly vacuolized. In extreme cases, the basal lamina was detached from the tubule. In the intestinal bulb and hindgut, high levels of histamine (4 g/kg) decreased the density of reserve inclusion cells. Thus, this indicated that histamine had dose-dependent effect on the activity of digestive enzymes and the morphology of the intestine and hepatopancreas.

## Background

The Chinese mitten crab (*Eriocheir sinensis*) is a native freshwater crab throughout the eastern region of China (Sui et al. 2009). Due to its delicate flavor and high nutritional value, the artificial culture production of this species in China has significantly increased from 8000 tonnes in 1991 to approximately 750,000 tonnes in 2015 (China Fisheries Yearbook 2015). During the culture of the Chinese mitten crab (*E*. *sinensis*) in China, trash fish and fish meal is usually provided as feed or feedstuffs, which decay rapidly due to improper processing, such as long-distance transport, high temperature of fish meal preparation, bad condition of storage and so on (Edwards et al. 2004; Wu et al. 2007). Stale trash fish contain high level of biogenic amines, such as histamine. Histamine is one common biogenic amine, formed by the decarboxylation of l-histidine, and has a high detection rate in fishmeal and seafood sold in many countries, including India, Thailand, the Philippines, and the Netherlands (Kennedy and Karunasagar 2004; Tao et al. 2011; Lee et al. 2015). The presence of this compound in feed or feedstuffs at high levels (exceeding 0.2–0.5 g/g) is considered to be toxic to the human and when ingested it caused gastric diseases in chickens (Harry and Tucker 1976), decreased weight gain and delayed sexual maturity in the aquatic animals (Yang et al. 2010). Therefore, histamine is used as a quality criterion for fish meals and also as an important safety indicator for food (Ricque-Marie et al. 1998; Tao et al. 2011).

At present, the effect of dietary histamine on digestive physiology of aquatic animals is still unclear. The main rout of dietary uptake is the digestive system, and the secretion of digestive enzymes and integrity of intestine and hepatopancreas are directly relevant to food intake and assimilation (Icely and Nott 1992; Simon 2009). When fed diets supplemented with histamine at 2 g/kg, a decline in feed consumption in the rainbow trout (*Oncorhynchus mykiss*) occurred (Fairgrieve et al. 1998). Whereas dietary histamine supplementation at 0.6–4.8 g/kg had no negative effects on feed consumption and feed conversion ratio in the blue shrimp (*Litopenaeus stylirostris*) (Tapia-Salazar et al. 2004). The divergence may be due to the varying ability of dietary histamine digestion among aquatic animals. But the reports are limited on the histopathology and enzymology of the digestive system after feeding dietary histamine in aquatic animals. Fairgrieve et al. (1994) reported that histamine supplementation of 2 g/kg caused intestinal damage in rainbow trout, including distended stomachs and stomach erosion. Watanabe et al. (1987) documented the effect of histamine on the formation of gastric lesions in rainbow trout (Watanabe et al. 1987; Fairgrieve et al. 1994). However, no further information about the effect of histamine on digestive physiology can be found in any other aquatic animals.

Digestive enzyme activities of crustaceans are important physiological parameters that reflect the digestive physiology of a crustacean and were suggested as predictors of potential feed utilization and growth differences (Rungruangsak-Torrissen 2001). The intestinal tract and the digestive gland (hepatopancreas) are the main sites of digestive enzyme secretion and also the first barriers against the interference of the whole organism (Pawert et al. 1996; Opstvedt et al. 2000; Lin and Luo 2011). However only few studies have combined molecular and biochemical procedures for describing the relationship between the transcription of the gene of a particular digestive enzyme and its activity (Muhlia-Almazán et al. 2003; Alvarez-González et al. 2010; Galaviz et al. 2012). Meanwhile, studies integrating the digestive system with expression and activation of digestive enzymes are scarce. In order to understand the relationship between tryptase and dietary histamine, tryptase mRNA and tryptase activity were determined. The aim of the current study was to provide further information about the effects of dietary histamine on growth, digestive enzyme activities and morphology of intestine and hepatopancreas in the Chinese mitten crab *Eriocheir sinensis*. It is the first report on the effect of histamine on the digestive physiology in aquatic animals.

## Methods

### Experimental diets

A basal (non-supplemented) diet was formulated Table 1, to meet the nutritional requirements of the Chinese mitten crab (*Eriocheir sinensis*) as recommended by Wu et al. 2010. A series of test diets was prepared by supplementing the basal diet with 1, 2, 4 g/kg of histamine. Diet stability was tested by immersing 5 g of diet samples in freshwater 26 °C (35 g/L) for 1 h according to Aquacop (1978) using six replicates for each diet. Dietary histamine content was determined by HPLC before and after the leaching test (Table 2).

**Table 1:** Control diet composition

Ingredients	%
Casein	41
Cellulose	6
Vitamin mix	2
Vc (90 %)	0.5
Ve (50 %)	0.1
Cholesterol	0.5
Inositol	0.6
Choline chloride (50 %)	1
Mineral mix	3
Yeast extractant	4
Betaine	0.15
Glycin	0.5
Soy lecithin (90 % phospholipid)	3
Dextrin	29.65
CMC	4
Fish oil	4

**Table 2:** Histamine concentration in the experimental diets (g/kg as free base)

Histamine supplemented	Before leaching	After leaching
0	4.40 ± 0.87	0
1	135.62 ± 3.17	117.14 ± 19.87
2	244.79 ± 10.15	215.47 ± 9.31
4	716.01 ± 23.54	470.70 ± 20.94

### Feeding trial

In the experiment, *Eriocheir sinensis* (25.98 ± 2.40 g initial body weight) were obtained from a commercial crab farm of Chongming Island in Shanghai and acclimated at 24.6 ± 0.75 °C, pH 8.39 ± 0.06, NH4^+^ 0.1–0.2 mg/L, nitrite <0.005 mg/L, under a photoperiod of 12L/12D for 1 week. Sixty individuals for each group were selected randomly and there were six tanks for each diet. Only intermolt crabs were used in experiments and a feeding trial was conducted in a recirculating water system for 28 days. The crabs were fed twice a day at 9:00 and 16:00. Feeding rate was calculated initially as 10 % of total body weight daily and the amount was then adjusted according to feed consumption in each tank to minimize the amount of uneaten feed. Faeces were removed by a siphon prior to the feeding the next morning (08:00 hours).

### Sampling for digestive enzymes and histological examination of crab tissues

Before sampling, the crabs were deprived of food for 24 h. Ten crabs were taken randomly after 7, 14, 21 and 28 days of each treatment. Hepatopancreas and intestinal tract tissue were removed from each crab. Part of the tissues was applied to determine the activities of digestive enzymes, another part was used for histological examination and analysis of tryptase mRNA hepatopancreas. The samples were stored at −80 °C until enzymes were assayed.

### Enzyme assays

A weighed (200–400 mg) hepatopancreas and intestinal tract tissue were homogenized in 10 volumes (W/V) of ice-cold distilled water for 5 min. Homogenates were centrifuged at 10,000 r/min for 30 min at 4 °C. The supernatant was separated and stored at −80 °C until digestive enzymes were assayed. All enzyme assays were conducted within 24 h after extraction.

Amylase activity and lipase activity were assayed by commercial kit (Nanjing Jiancheng Bioengineering Institute, Nanjing, China).

Tryptase activity was measured by casein hydrolysis (modified by Pan and Wang 1997). All samples were assayed six times and the blanks in triplicate. One enzyme unit was defined as the amount of enzyme that catalyzed the release of 1 lg of product per min under the assay conditions. Protease, amylase, and lipase activities were expressed as enzyme activity per mg protein (U/mg prot). The total soluble protein concentration was determined by the method of Bradford (1976) using bovine serum albumin as a standard.

### Histological examination

Six crabs were randomly sampled for gross histological examination of the hepatopancreas and intestinal tract (including midgut, intestinal bulb and hindgut) at the beginning (0 day) and end of the experiment (28 days). All tissue samples were fixed immediately in Bouin’s fluid. After cleansing in ethanol the tissues were dehydrated in ethanol, treated with xylene, embedded in paraffin wax, sectioned in 5 μm slices, and then stained with haematoxylin, eosin. Examination was done by light microscopy (Leica DM2500) with a photomicrographs attached to a computer with Motic Images software (Leica Application Suite V3.3.0).

### Quantitative real-time PCR (RT-PCR) analysis of tryptase gene expression

Total RNA isolation and reverse transcription were performed according to Muhlia-Almazán et al. (2003) and Galaviz et al. (2012). Primers for amplification of tryptase were designed based on the sequence of a complete ORF of *E*. *sinensis* tryptase (GenBank accession no. EF530707), β-actin was used as the house-keeping gene. For details see Table 3, RT-PCR reaction conditions was performed for 40 cycles, under the following cycling conditions: 95 °C for 30 s, 95 °C for 5 s, 60 °C for 20 s, and fluorescent reading.

**Table 3:** Tryptase genes of *Eriocheir sinensis* and their corresponding PCR primers used for quantitative real time PCR

Primers	Sequence
Forward primer	5′-TGATGAGGGTAACGAGCAGG-3′
Reverse primer	5′-TTGAAGGTCAGAGGGGAGGA-3′
β-Actin-F	5′-GCATCCACGAGACCACTTACA-3′
β-Actin-R	5′-CTCCTGCTTGCTGATCCACATC-3′

### Statistical analysis

The data (mean ± SEM) from the control and treatment groups were subjected to a one-way analysis of variance (ANOVA) and compared using Tukey’s test (p < 0.05). All of the statistics were performed using the SPSS 11.0 software package.

## Results

### Changes in body mass of the *E*. *sinensis* during the course of the experiment

There was no significant difference in the body mass between the beginning and the end of experiment (*p* > *0*.*05*) (Table 4).

**Table 4:** Changes in body mass of the *E*. *sinensis* during the course of the experiment

Group	Initial (g)	At the end of experiment (28 days)
Control (0 g/kg)	26.66 ± 2.18	27.65 ± 2.52
1 g/kg	26.46 ± 1.91	26.82 ± 2.03
2 g/kg	26.20 ± 2.78	26.53 ± 2.01
4 g/kg	26.17 ± 1.69	27.54 ± 1.47

### Changes of digestive enzyme activities in the hepatopancreas and intestinal tract tissue during the period of experiment

#### In the hepatopancreas

**Fig. 1:**
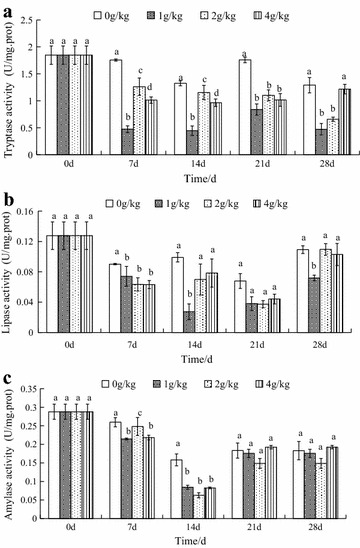
Effects of dietary histamine supplementation on the activities of digestive enzymes in the hepatopancreas in *Eriocheir sinensis* (**a** tryptase activity, **b** lipase activity, **c** amylase activity). The values are mean ± SD from three individual samples. Using one-way ANOVA, compared with the control, data at the same elapsed time with *different letters* are significantly different (p < 0.05) among treatments

The results indicated that dietary histamine inhibited tryptase activity in the hepatopancreas during the experimental period, as shown in Fig. 1A. In crabs fed with diet supplemented with histamine for 28 days, tryptase activity significantly decreased among the different histamine treatments in each time point compared to the control, respectively (Fig. 1A) (*p* < *0*.*05*), with the exception of the 4 g/kg histamine group, that showed a slight recovery on day 28. The tryptase activity of the group that received 1 g/kg histamine decreased by 74.35, 75.87, 54.59, 74.41 % (*p* < *0*.*05*) as compared to the control. Strangely, tryptase activity in the control on day 14 and 28 showed slightly decreased.

All the histamine treatments showed significantly decreased lipase activity on day 7 as compared to the control group (p < 0.05), but there were no significant differences among the different histamine treatments. When compared to the control, The lipase activity of the group that received 1 g/kg histamine decreased by 71.88 % compared to the activity of the control group on day 14. On days 21 and 28, lipase activity in almost all groups was recovered to levels similar to those of the control group, with the exception of the 1 g/kg histamine group, that retained low lipase activity, compared to the control (Fig. 1B). As seen in Fig. 1B, 1 g/kg histamine treatments showed significantly decreased by 34.77 % as compared to the control on day 28.

In each histamine-treated group, amylase activity was significantly decreased on day 7 and 14 as compared to the control and the values reached their lowest on day 14 (p < 0.05). As seen in Fig. 1C, amylase activity in all groups exhibited a slight recovery on day 21 and 28 and no difference was found among the histamine treated groups and the control (p > 0.05).

#### In the intestinal tract

**Fig. 2:**
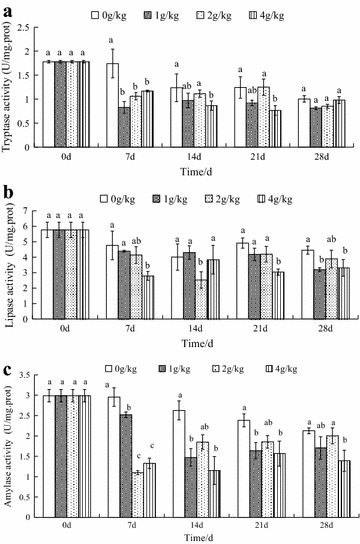
Effects of dietary histamine supplementation on the activities of digestive enzymes in the intestinal tract in *Eriocheir sinensis* (**a** tryptase activity, **b** lipase activity, **c** amylase activity). The values are mean ± SD from three individual samples. Using one-way ANOVA, compared with the control, data at the same elapsed time with *different letters* are significantly different (p < 0.05) among treatments

As seen in Fig. 2A, tryptase activity was reduced by all histamine treatments in each time point. On day 7, 14 and 21, the 4 g/kg histamine treatments showed tryptase activities significantly decreased by 32.89, 29.97, 38.35 % compared with the control group (*p* < *0*.*05*). At the end of experiment, tryptase activity showed a recovery and no difference between each histamine treatments and control group. Strangely, tryptase activity was also inhibited slightly in each control group on day 14, 21 and 28.

As seen in Fig. 2B, lipase activity decreased and then increased in all histamine treatments as compared to the control with the time elapsed. When compared to the control, 1 g/kg (71.68 % of control), 2 g/kg (63 % of control), 4 g/kg (56.72 % of control) histamine treatments showed lipase activities significantly decreased to the lowest value on days 28, 14 and 7, respectively (*p* < *0*.*05*).

As seen in Fig. 2C, amylase activity was inhibited by all histamine treatments in each time point compared to the control group. There were significant differences on day 7 (*p* < *0*.*05*). When compared to the control, amylase activity was significantly decreased by both 1 and 4 g/kg histamine on days 14 and 21. In the group receiving the high histamine concentration (4 g/kg), amylase activity decreased significantly by 34.34 % of control on day 28.

### Histamine induced changes in tryptase mRNA expression in the hepatopancreas

**Fig. 3:**
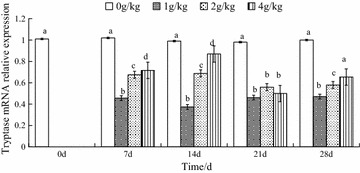
Effects of dietary histamine supplementation on the tryptase mRNA expression in the hepatopancreas in *Eriocheir sinensis*. The values are mean ± SD from three individual samples. Using one-way ANOVA, compared with the control, data at the same elapsed time with *different letters* are significantly different (p < 0.05) among treatments

At each time point, all the histamine-treated groups showed lower tryptase mRNA relative expression than that of the control group and had significant difference from the control (*p* < *0*.*05*). There was no difference to compare the tryptase mRNA levels among the control groups at time 0, 7, 14, 21 and 28 days. On day 7, 14 and 28, tryptase mRNA relative expression in the histamine-treated groups correlated positively with the histamine concentration (*p* < *0*.*05*). There were no marked differences among the histamine treatments on day 21. The 4 g/kg histamine treatments exhibited a slight recovery of tryptase mRNA relative expression on days 28 compare with the control (*p* > *0*.*05*) (Fig. 3).

### Histological study

**Fig. 4:**
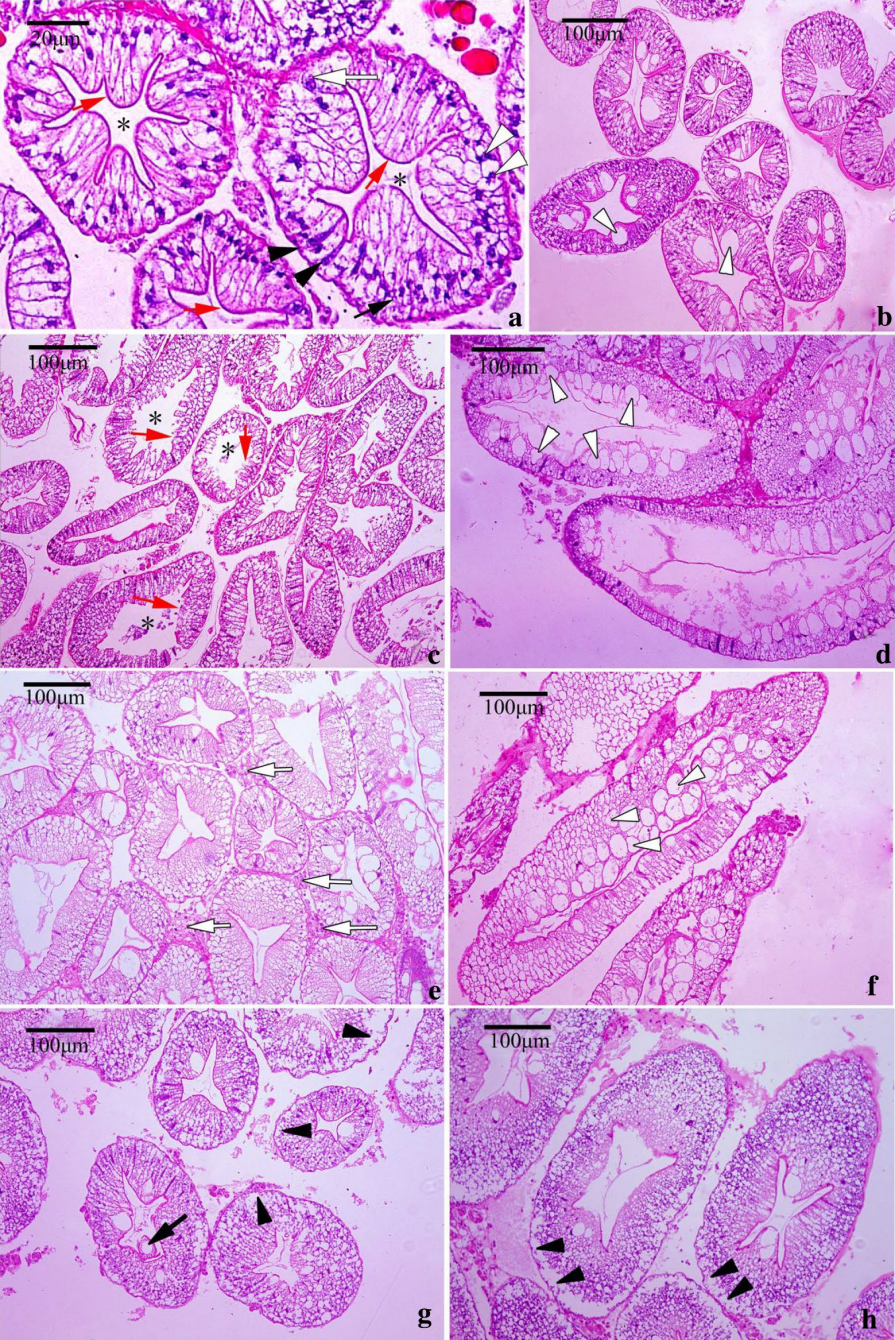
Histological observations of hepatopancreas from Chinese mitten crab fed with diet containing histamine after 28 days. **a**, **b** Hepatopancreas of control crabs (histamine 0 mg/kg) showing complete structure of different cells in the control; Hepatopancreas exhibited normal star-shaped lumina (L) (*asterisk*) and intact brush border (*red arrow*). The four cell types:B cell (*white arrowhead*), F-cell (*black arrowhead*), R-cell (*black arrow*), E-cell (*white arrow*) were clearly visible. **c**, **d** Crabs fed diet containing histamine 1 g/kg, loss of star-shape lumina (*asterisk*) and brush border (*red arrow*) damaged and the number of B-cell increased (*white arrowhead*); **e**, **f** crabs fed diet containing histamine 2 g/kg, all cells were highly vacuolized (*white arrowhead*), haemocytes occurred among the tubules (*white arrow*). **g**, **h** One vacuole were released into the tubule lumen (*black arrow*), basal lamina were detached from tubule (*black arrowhead*) and cell boundary was unclear observed

Figure 4a–h show the transverse and longitudinal section of the tubules through the medial region of hepatopancreas. Hepatopancreas from a crab unfed histamine exhibited normal star-shaped lumina (L) (Fig. 4a, b). The cell types (B, F and R) described previously (Al-Mohanna and Nott 1989) were clearly visible. B cells were distinguished by the large and single digestive vacuole, F cells, between the B and R cells, were more darkly stained. R cells were the most abundant cell in the control group (Fig. 4a). A brush border (Bb) is evident on the luminal surface of the cells and remains intact (Fig. 4a, b). The tubules in the hepatopancreas of the control crabs were closely arranged to each other with basal laminae surrounding each tubule (Fig. 4a, b).

Hepatopancreas of crab fed the three different histamine-supplemented diets revealed some common abnormalities (Fig. 4c–h). It includes unusual enlargement of the lumina (Fig. 4c, d), loss of the star-shaped structure and lesion of brush border (Fig. 4c, d). The number of B (secretory) cells present in the tubular epithelium were significantly increased (Fig. 4d). The hepatopancreas of crabs fed a diet containing histamine 2 g/kg exhibited haemocytes among the tubules (Fig. 4e) and high vacuolization. In extreme cases, the cells take on a foamy appearance (Fig. 4f). Several B-cells were bulged into the lumina (Fig. 4f). One vacuole had been released into the tubule lumen (Fig. 4g). The detachment of basal lamina from tubule and loss of structural integrity were commonly observed (Fig. 4g, h). A higher number of R-cells were present in the tubule and the cell boundaries were difficult to observe (Fig. 4g, h).

**Fig. 5:**
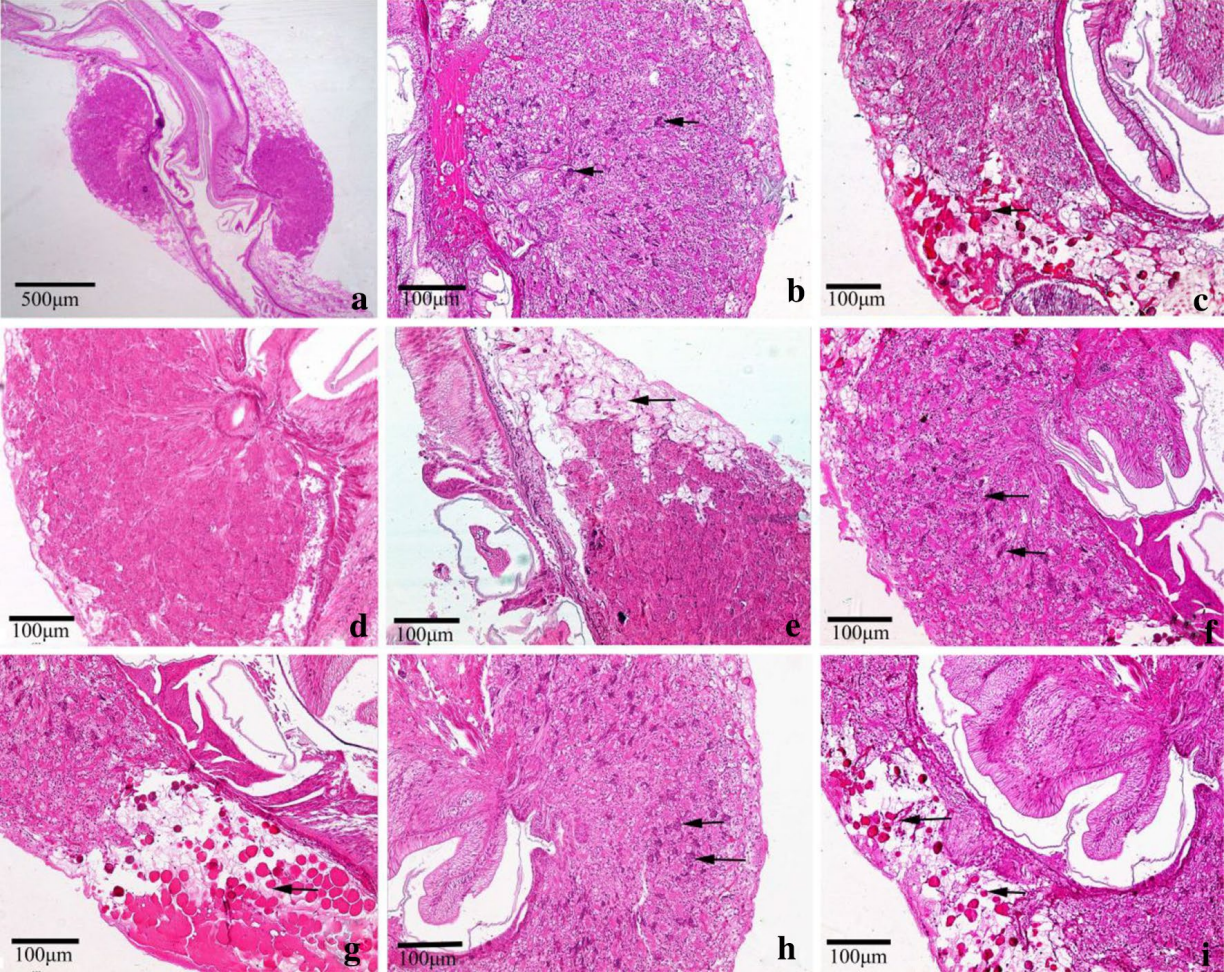
Histological observations of intestinal bulb from Chinese mitten crab fed with diet containing histamine after 28 days. **a** Longitudinal section of intestinal bulb showing the gross structure in the control (*arrow*). **b** The basophilic nucleus were visible in the intestinal bulb in the control (*arrow*). **c** Adjacent to the intestinal bulb, a reticular appearance and reserve inclusion cells were present (*arrow*). **d**, **e** Basophilic nucleus were hardly visible and reserve inclusion cells were fewer in 1 g/kg histamine treatments (*arrow*). **f**, **g** The number of basophilic nucleus and reserve inclusion cells increased in 2 g/kg histamine treatments (*arrow*). **h**, **i** The number of basophilic nucleus increased and reserve inclusion cells decreased in 4 g/kg histamine treatments (*arrow*)

The intestinal bulb, connecting midgut and hindgut had an enlargement of the serosa, which was typically spherical and the basal part containing the basophilic nuclei was long and filamentous (Fig. 5a, b). Adjacent to the intestinal bulb, the cells had a reticular appearance and had red colored cytoplasmic granules–reserve inclusion cells (Fig. 5c). Morphological analyses of the intestinal bulb from crabs fed dietary histamine for 28 days showed histopathologic alterations at all tested concentrations of histamine with the nuclei and reserve inclusion cells (Fig. 5d–i). The nuclei of the intestinal bulbs and the reserve inclusion cells were scarce or disappeared in 1 g/kg histamine treatments (Fig. 5d, e). As seen in Fig. 5f, g, the number of nuclei and reserve inclusion cells increased in 2 g/kg histamine treatments. The regions of the nuclei were larger and reserve inclusion cells were fewer in 4 g/kg histamine treatments than those in the control (Fig. 5h, i).

**Fig. 6:**
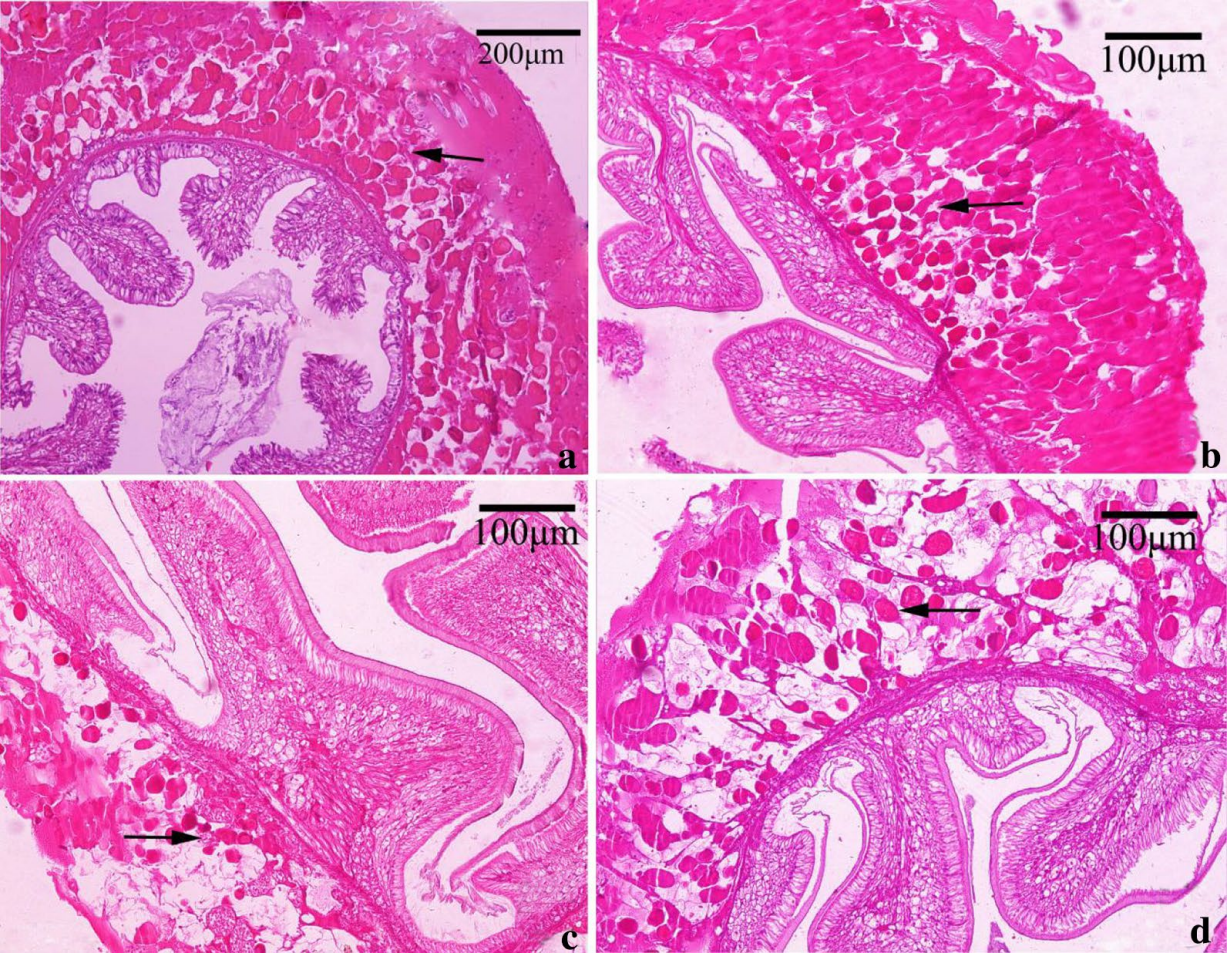
Histological observations of hindgut from Chinese mitten crab fed with diet containing histamine after 28 days. **a** Cross section of hindgut in the control, eosinophilic cells occurred (*arrow*), **b** cross section of hindgut in 1 g/kg histamine treatments, reserve inclusion cells became more dense (*arrow*) compared to that in the control, **c** and **d** cross section of hindgut in 2 and 4 g/kg histamine treatments, respectively, reserve inclusion cells became sparse (*arrow*)

The wall of the hindgut consists of the tunica mucosa, tunica submucosa, tunica muscularis and tunica serosa. The well-developed tunica serosa contained loose connective tissue, adipose tissue, blood sinuses and nerves (Babu et al. 1982). Interestingly, a large number of eosinophilic cells–reserve inclusion cells were also found in the tunica serosa in the control (Fig. 6a). After feeding dietary histamine for 28 days, the number of the reserve inclusion cells were changed (Fig. 6b–d). Specifically, the reserve inclusion cells were more dense in 1 g/kg histamine treatments when compared to the control (Fig. 6b), while the cells became more sparse in 2 and 4 g/kg histamine treatments compared to the control or the 1 g/kg histamine group (Fig. 6c, d).

## Discussion

### Digestive enzyme

Digestive enzyme activities of crustaceans are important physiological parameters that reflect the digestive physiology of a crustacean and are closely related to heredity, environment, and food habit. Digestive enzymes of crabs are mainly provided and excreted by the digestive tract (consisting of stomach and intestine) and the digestive gland (hepatopancreas).

In the present study, we have demonstrated that the activities of digestive enzymes decreased significantly at first (*p* < *0*.*05*) and then increased or finally slightly recovered in the hepatopancreas and intestinal tract after feeding dietary histamine for 28 days. It is suggested that crabs had the capacity to adapt their digestive physiology in response to changes in their dietary histamine to some extent. A similar observation was reported by Opstvedt et al. (2000) and Lin and Luo (2011). However, it seems to me that just by keeping the crabs under their experimental conditions and standard diet, specific changes occur in the acitivites of the tested enzymes in both the intestinal tract and hepatopancreas. Histamine appears to speed up the effect.

As seen in Fig. 1, the results indicated histamine could have a more profound effect on tryptase activity than lipase and amylase activities in the hepatopancreas. Watanabe et al. (1987) reported that dietary histamine can stimulated the secretion of proteases of rainbow trout; Leng et al. (2003) reported that the addition of histamine in the diet of early weaned piglets could increase the secretion of pepsin and improve trypsin activity. It is possible that histamine was related to protease activities. In rabbits, histamine (10^−3^ M) was found to stimulate pancreatic trypsinogen secretion from in vitro isolated rabbit pancreas preparations, but high concentrations (10^−2^ M) of histamine are not unlike (Liebow and Franklin 1982). A similar effect of histamine was also found in the growth of blue shrimp (Tapia-Salazar et al. 2004), immunity of crabs (Zhao et al. 2012) and liver function of rabbits (Tripathi et al. 2012). In these animals, the maximum effect of histamine is not the highest concentration but the lower concentration. Watanabe et al. (1987) reported that the level of dietary histamine stimulating the secretion of protease was 700 µg/g, Leng et al. (2003) found only 60 μg/kg histamine could stimulate trypsase and too low or too high level of histamine had no effect on the tryptase activities. Therefore, different animals may have their own special sensitivity to different level of histamine. Some researchers reported that high mortalities have been observed in poultry and mysis with dietary histamine supplementation (Harry et al. 1975; Osuna 1985; Yang et al. 2010), while histamine supplementation had no influence on shrimp and rainbow trout mortality (Fairgrieve et al. 1998; Tapia-Salazar et al. 2004), what’s more, histamine improves survival of mice (Hornyak et al. 2005). Similar species differences in sensitivity to histamine have been demonstrated by mammals in studying gastric acid and pepsinogen secretion (Liebow and Franklin 1982), but no explanation was found for a correlation between histamine and sensitivity among species.

Strangely, tryptase activity in the control on day 14–28 showed slightly decreased in the hepatopancreas and intestine. This changes of tryptase activity may be a physiological adaptability of motling and feeding, because the time is close to the pre-molt stage in the end of the experiment. Lin et al. (2013) found that the food consumption and tryptase activity in *Portunnus trituberculatus* decreased at 10–12 days after molting, which is close to the next pre-molt. In the present study, it is the most difficult to keep all the crabs in the same molting stages, and how to avoid the slight decrease of tryptase activity in the control would be the next challenge.

By Fluorescent quantitation PCR, the results indicated that tryptase mRNA relative expression was basically consistent with that of traditional enzyme assays, with the exception of the 2 g/kg histamine treatment on the 7th and 14th day. That suggested that the time for trypsin synthesis and enzymatic activity could be specific, would depend on the levels of exogenous feeding histamine. Thus, similar results were also observed in the effects of starvation on the trypsin activity in *Lateolabrax japonicus*, which suggested that the changes of tryptase activity not only due to its secretory volume, but also its external environment (Muhlia-Almazán et al. 2003; Galaviz et al. 2012).

### Histological examination

The hepatopancreas of crustaceans has long been thought to function not only as a site for secretion of digestive enzymes, but also as a center for another multitude of metabolic processes including protein synthesis and detoxification (Al-Mohanna and Nott 1989; Pinho et al. 2003; Xiao et al. 2014). The epithelia of the intestinal tract and digestive gland (hepatopancreas) are the first barriers against the poisoning of the whole organism (Pawert et al. 1996).

The results of the histological observations are summarized in Figs. 4, 5 and 6, which showed that, even with the lowest histamine levels, serious alterations were observed in the hepatopancreas, moderate alterations in the hindgut and intestinal bulb, and no alterations in the midgut. Indeed, this difference may be related to the main site of histamine to metabolize. Histamine is derived from l-histidine by histidine decarboxylase after decarboxylation. Shiozaki et al. (2003) showed the exogenous histamine in rainbow trout (*Oncorhynchus mykiss*) with oral administration was easily detoxified in digestive organs and histamine was carried to the liver (Shiozaki et al. 2003); moreover, the important metabolic enzymes of histamine were detected in the intestine in many fishes, such as eel (Holstein 1975), mackerel, tuna and yellowtail (Matsumiya and Otake 1981). It is possible, therefore, that a part of histamine can be metabolized in the intestinal tract, especially the midgut and the other part of histamine and its metabolites could be stored or transported to hepatopancreas. Unfortunately, histamine metabolites were not analysed in the present experiment.

Generally speaking, there are four cell types (E-cells, F-cells, B-cells and R-cells) comprising the digestive surface of the hepatopancreas tubules. F-cells are the source of digestive enzymes, and R cells are responsible to store lipid, glycogen and debris. When debris accumulates, the R cells transform into B cells. Berillis et al. (2013) reported B-cells that are not lost to the tubule lumen through holocrine secretion undergo a restitution cycle, during which the cells appear as F-cells before developing again into B-cells. B cells play a role in absorption and digestion, and more important function is to remove accumulated debris and waste products by sloughing off into the lumina of the proximal tubules (Al-Mohanna and Nott 1989; Pinho et al. 2003; Xiao et al. 2014). Pinho et al. (2003) and Xiao et al. (2014) reported that the increase of B-cells indicated that the high rate of synthesis and release of digestive enzymes accelerate the mobilization of energy in hepatopancreas to adapt to stress (Pinho et al. 2003; Xiao et al. 2014). The present study showed that 1 g/kg histamine increased the number of B-cells in hepatopancreas on the 28th day. In addition, the number of R-cells and B-cells in 2 g/kg histamine group were more than that in 1 g/kg histamine group. Thus, this can partly explain why amylase and lipase activities exhibited a slight recovery on the 28th day. However, in 4 g/kg histamine treatment vacuole were released into the tubule lumen, B-cells were rare and cell boundary was difficult to be observed, which suggested that high level of histamine damaged normal structure of the hepatopancreas. Of course, changes in the lumen, R-, B- and F-cells maybe linked with food consumption and molting (Berillis et al. 2013; Simon and Jeffs 2008).

The intestinal bulb were the transition of midgut and hindgut and had similar organization structure both midgut and hindgut (Fang et al. 2002), and the hindgut in *Eriocheir sinensis* played a part in peristalsis, temporary storage and discharge of fecal (Fang et al. 2002). Unfortunately, the function of the intestinal bulb was still unclear. Our results showed that there were a large number of reserve inclusion cells in the intestinal bulb and the hindgut, and the levels of histamine had relationship with the number of those cells (Fig. 5d–i). In *Panulirus stimpsoni*, we found similar shape of red cytoplasmic granules in the digestive tract and the granules showed PAS and bromophenol blue positive reaction, that is rich in protein and lipid, suggesting this granules were close relations with protease and lipase enzyme (Jiang and Yan 2009). Zhao et al. 2014 reported that this eosinophilic particular material showed mast cells positive staining in *Eriocheir sinensis* and found morphological and staining properties of eosinophilic particular material in the hindgut are quite similar to the mast cells in digestive tract of zebrafish (Iván et al. 2007; Zhao et al. 2014; Sfacteriaa et al. 2015). Similarly Reite (1996) stated that eosinophilic granule cells (EGC) are homologous of mast cells both in the structural and functional properties to mammalian mucosal mast cells, moreover mast cells were the major source of the body’s histamine (Reite 1996). Further, it appears that the digestive tract of many fish species is the richest source of mast cells (Sfacteriaa et al. 2015). Thus, this can partly explain how histamine influenced the number of reserve inclusion cells to change the activity of tryptase and lipase. However, the relationship of histamine and digestive function in invertebrates and fish were unclear, the detail explanations need to further research in the long run.

## Conclusions

During a 28-days feeding experiment, we found histamine supplementation did not have any effect on growth of the Chinese mitten crab (*Eriocheir sinensis*). Further, the activities of digestive enzyme in the hepatopancreas and intestinal tract decreased significantly at first (days 7 and 14) (p < 0.05) and then increased or slightly recover in the end. On day 7, 14 and 28, tryptase mRNA relative expression in the histamine-treated groups correlated positively with the histamine concentration (p < 0.05). Histopathological analyses showed, even with the lowest histamine levels, serious alterations were observed in the hepatopancreas, moderate alterations in the hindgut and intestinal bulb, and no alterations in the midgut. These results will give the guidline for the suitable quantity of trash fish used in the aquaculture of the Chinese mitten crab (*Eriocheir sinensis*), especially in China, and lay the foundation for the further digestive physiology of histamine in crustacean.
